# ^3^Cat-3/MOTS Nanosatellite Mission for Optical Multispectral and GNSS-R Earth Observation: Concept and Analysis

**DOI:** 10.3390/s18010140

**Published:** 2018-01-06

**Authors:** Jordi Castellví, Adriano Camps, Jordi Corbera, Ramon Alamús

**Affiliations:** 1 Teoria del Senyal i Comunicació (TSC), Universitat Politècnica de Catalunya (UPC), 08034 Barcelona, Spain; camps@tsc.upc.edu; 2 Institut Cartogràfic i Geològic de Catalunya (ICGC), Parc de Montjuïc, 08038 Barcelona, Spain; jordi.corbera@icgc.cat (J.C.); ramón.alamus@icgc.cat (R.A.)

**Keywords:** GNSS-R, soil moisture, downscaling, mission analysis, earth observation

## Abstract

The ^3^Cat-3/MOTS (3: Cube, Cat: Catalunya, 3: 3rd CubeSat mission/Missió Observació Terra Satèl·lit) mission is a joint initiative between the Institut Cartogràfic i Geològic de Catalunya (ICGC) and the Universitat Politècnica de Catalunya-BarcelonaTech (UPC) to foster innovative Earth Observation (EO) techniques based on data fusion of Global Navigation Satellite Systems Reflectometry (GNSS-R) and optical payloads. It is based on a 6U CubeSat platform, roughly a 10 cm × 20 cm × 30 cm parallelepiped. Since 2012, there has been a fast growing trend to use small satellites, especially nanosatellites, and in particular those following the CubeSat form factor. Small satellites possess intrinsic advantages over larger platforms in terms of cost, flexibility, and scalability, and may also enable constellations, trains, federations, or fractionated satellites or payloads based on a large number of individual satellites at an affordable cost. This work summarizes the mission analysis of ^3^Cat-3/MOTS, including its payload results, power budget (PB), thermal budget (TB), and data budget (DB). This mission analysis is addressed to transform EO data into territorial climate variables (soil moisture and land cover change) at the best possible achievable spatio-temporal resolution.

## 1. Introduction

The emergence of small satellites, and in particular the CubeSat standard [1], has opened up new ways of exploiting space [2]. Future projections foresee thnat in the next 5–10 years most satellites will be small satellites of less than 50 kg (nano and microsatellites) [3]. The inherent strategy behind the small satellites approach has allowed for ES (Earth Science) missions, such as ^3^Cat-3/MOTS (3: Cube, Cat: Catalunya, 3: 3rd CubeSat mission/Missió Observació Terra Satèl·lit), at an affordable risk and cost [4]. However, small satellites present limitations and vulnerabilities associated with their low cost philosophy. In order to overcome a hostile space environment, both the behavior of the different subsystems and that of the components need to be accurately studied [5].

The mission statement of ^3^Cat-3/MOTS is based on three pillars: viability (budget, human resources, and know-how), feasibility (technology readiness), and desirability (usefulness of the results). These three concepts converge in ^3^Cat-3/MOTS with an innovative combined optical/ Global Navigation Satellite Systems Reflectometry (GNSS-R) [6,7] payload and data fusion solution for high-resolution soil moisture mapping. Since the concept of innovation is linked to a limited time window, the combination of the knowledge provided by the Institut Cartogràfic i Geològic de Catalunya (ICGC) in the field of satellite imagery applied to the management of the Catalan territory, and the experience of the Universitat Politècnica de Catalunya-BarcelonaTech (UPC) in the field of Remote Sensing, in the development of previous small satellite missions (^3^Cat-1 [8], ^3^Cat-2 [9], and ^3^Cat-4 and ^3^Cat-5 as part of the FYS (Fly Your Satellite) program [10] and FSSCat (Federated Satellite System Catalunya) [11] respectively, both in the design phase), and in the access to facilities to test and qualify space hardware [12] define an optimum environment for ^3^Cat-3/MOTS.

The main goal of ^3^Cat-3/MOTS is to acquire multispectral imagery of the Earth in conjunction with GNSS-R data. The regions of interest (ROIs) for this mission will be land cover areas with high vulnerability and impact by climate change. The ICGC has defined a set of design requirements (Table 1) where the specific criteria for attitude control, exploitation, and data acquisition are detailed. In parallel to this main goal, the mission objectives can be listed as:

1. To identify the limit of current CubeSat technology in terms of spatial resolution and required power to accomplish a multispectral optical and GNSS-R space mission.
2. To evaluate the feasibility of using Commercial off the Shelf (COTS) optical equipment in space to achieve ^3^Cat-3/MOTS’s mission requirements.
3. To acquire multispectral images from the visible to the near infrared (400 nm to 870 nm) with a spatial resolution better than 30 m and swath wider than 30 km with a signal-to-noise ratio (SNR) better than 30 dB in each band.
4. To achieve a revisit time of less than 10 days over the Catalan territory to properly respond to territorial changes.
5. To perform data fusion of the observables acquired by both payloads: multispectral imagery from the optical sensor and L-band reflectometry data from the GNSS-R soil moisture mapping at 30 m resolution.

## 2. Materials and Methods

### 2.1. Orbit Selection

The main tradeoff that drives the mission concept is between spatial resolution and mission lifespan: the limited size of the CubeSat does not allow for a large optical system; thus, in order to achieve a good spatial resolution, a low orbit is recommended. The ideal orbit is therefore an inclined orbit at ~500 km height and 55° inclination so as to cover most areas of interest (urban regions) and for orbit stability purposes [13]. A common choice for Earth Observation (EO) missions with optical sensors is a Sun-Synchronous Orbit (SSO) with a determined Local Time of Ascending Node (LTAN) designed to acquire images of the ROI with a constant illumination. However, the proposed orbit is not an SSO since the nature of the mission is strongly focused in the region of Catalonia, Spain, and the chosen orbit reduces the revisit time. In practice, the final orbit will be the best available one, taking into account that CubeSats are launched as a piggyback on a larger primary satellite that is the one that drives the orbit selection.

### 2.2. Platform Selection

The CubeSat standard allows for several configurations. Currently, there are 1U up to 6U CubeSats in orbit, although future missions with 12U and 27U units are already planned. After a detailed study, the 6U unit is the smallest CubeSat platform to fit the payload’s main requirements. Two arguments lead to this conclusion:

1. High spatial resolution is achieved by using sensors with a small detector size and long focal lengths. This discards 1U and 2U CubeSats in favor of 3U and 6U ones. In a 3U, most of the inner space would be used for the optical sensor and the optical train, leaving little space for the GNSS-R payload (antenna and microwave receiver) and all other satellite subsystems.
2. The electrical power to be supplied to the subsystems and payload of the ^3^Cat-3/MOTS cannot be supplied by a 3U CubeSat.

### 2.3. Preliminary Concepts and Simulation Configuration

One of the design guidelines of the mission has been the correct estimation of the satellite’s lifespan and orbit lifetime. There are several internal and external factors that reduce the lifespan of the satellite (charged particles, solar radiation, extreme temperature variations, batteries’ depth of discharge and number of charge/discharge cycles, aging of the electronic devices, etc.). On the other hand, there is the orbital decay, which is mostly due to atmospheric drag and is especially significant for low earth orbit (LEO) orbits. The reentry of the satellite is both inevitable and desirable, but only when the mission is finished. As with most CubeSats, the ^3^Cat-3/MOTS will not have orbital maneuver capabilities; therefore, the satellite’s lifespan and its orbit lifetime should be similar.

#### 2.3.1. Orbital Lifetime

**Figure 1:**
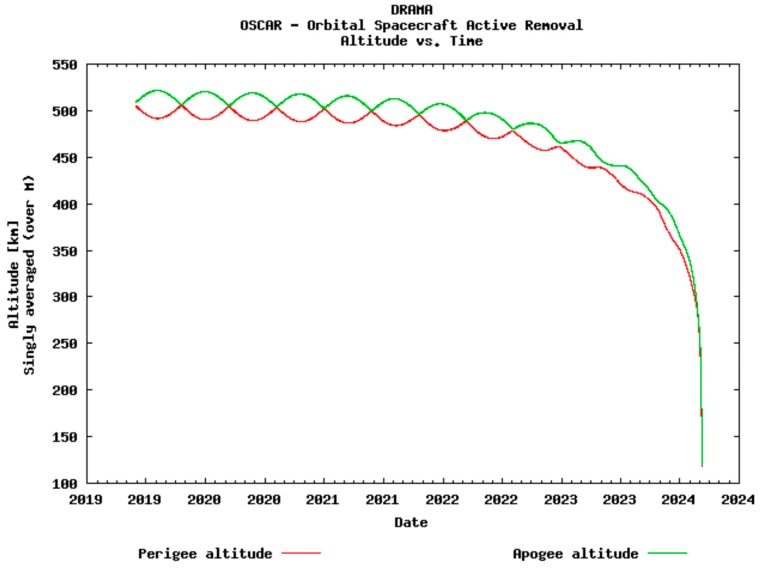
Apogee/Perigee Altitude history for the studied orbit (Start Year: 2019, Inclination: 55°, RAAN (Right Ascension of the Ascending Node): 8.32°, Arg. Peri.: 0°, Mean Anomaly 0°, and Area-to-Mass: 0.006 kg/m^2^). The initial orbital height for the simulation has been set to 500 km. Reentry will occur after 4.6 years, while the mission has an expected lifespan of 2 years. During this period, the changes in the orbital height are negligible for payload functioning purposes.

Given the characteristics of the optical payload and the pursuit of high spatial resolution images, a 500 km orbital height is assumed for simulation purposes in the present study. The simulation of the satellite’s orbital lifetime, the reentry time, and the orbital height fluctuations along the mission have been predicted using DRAMA-OSCAR Graphical User Interface V.2.1.0 [14] (Figure 1). The lack of any propulsion system and the need to comply with the recommendations [15] forces us to design an orbit with a reentry time under 25 years. The simulation performed considered a cross-sectional area equal to 0.01 m^2^ and a typical drag coefficient of 2 for LEO orbits [16], and it predicts a re-entry in 4.6 years. The re-entry survival analysis shows that no debris will reach the Earth’s surface and that all devices will disintegrate between 71 km and 78 km height (Table 2). The reentry time of 4.6 years is in compliance with the ^3^Cat-3/MOTS mission requirements of a lifespan and orbit lifetime of at least 3 years [17].

#### 2.3.2. Shielding

Radiation and high-energy particles destroy the electronic components of the satellite at a microscopic level (transistor size in 2017 is 7–5 nm [19]). There are several sources of radiation, and different techniques must be applied to protect platforms from their damaging effects:

1. Galactic Cosmic Rays (GCR) are high-energy charged particles that have originated outside our Solar system. Shielding is not effective to protect the platform against GCR.
2. Solar Energetic Particles (SEP) are electrons, protons, and heavy ions that have originated in the Sun. Also, gradual events accelerated by Coronal Mass Ejections (CME) and impulsive events from Solar flares present a risk to the satellite’s electronics.
3. Solar wind: Plasma of charged particles causing disturbances in the magnetosphere.
4. Radiation belts: Charged particles (protons and electrons) trapped by the Earth’s magnetic field.

**Figure 2:**
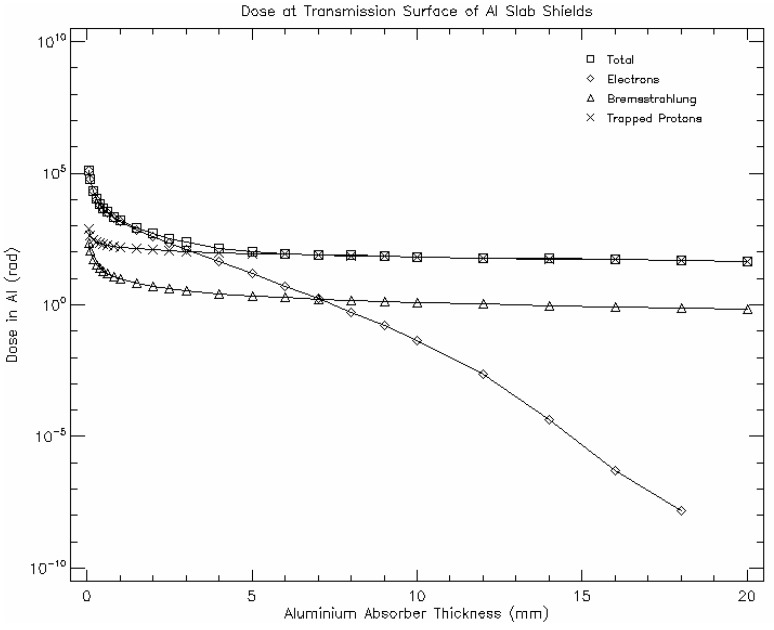
Accumulated radiation dose (rad) as a function of the shielding thickness. The main contributors to the Total Ionizing Dose (TID) include trapped protons and electrons and Bremsstrahlung protons. The total dose accumulated is efficiently reduced up to a 5 mm shielding thickness.

In order to secure the survivability of the satellite for at least 2 years, it is mandatory to implement some shielding around essential components, such as the payload, the OBC (On Board Computer), and the Communication System. The high-energy radiation is mainly blocked by the amount of mass (thickness of the shielding) of the impacted material. Aluminum layers have been thoroughly used as shielding, but Z-graded shields have also proven to perform with a reduced mass compared to Aluminum layers. The NASA Shields-1 technology demonstrator [20] seeks to test Z-graded shielding technology (a laminate of several materials with different atomic numbers, designed to protect against ionizing radiation) [21] in a CubeSat with the corresponding limitations in both mass and available room. SPENVIS [22] has been used to perform the radiation analysis, mainly focusing on trapped proton and electron fluxes, galactic cosmic ray fluxes, and damage equivalent fluencies for solar cells. Both trapped protons and electrons contribute to the Total Ionizing Dose (TID) along with Bremsstrahlung protons and Solar flare protons, but in LEO orbits, protons dominate over electrons in contribution to the TID [23], (p. 30). Shielding, therefore, will be essential to the survival of the mission, but given the high constraints of mass and size of the 6U CubeSat, the thickness of the shielding should be carefully adjusted to comply with its protective task for the duration of the mission with the minimum weight (Figure 2). A 3 mm Aluminum plate shielding (84.3 gr) minimizes both the radiation dose accumulated and the shield mass.

#### 2.3.3. Scheduler

**Figure 3:**
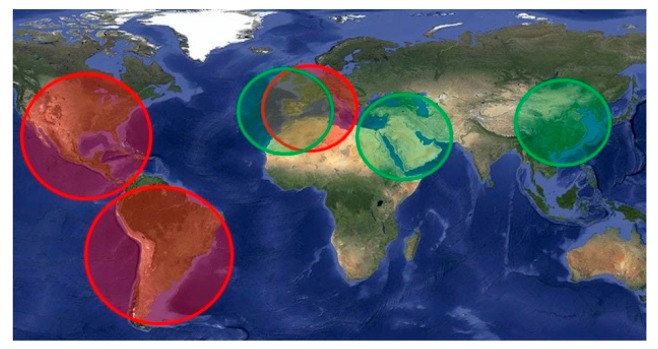
Target areas (TA) in red and ground stations (GS) in green considered for the simulation. The radii of the TAs (from left to right) are set to 3000 km, 3500 km, and 2000 km, respectively. The ground stations have a minimum elevation angle over the horizon of 10°.

**Figure 4:**
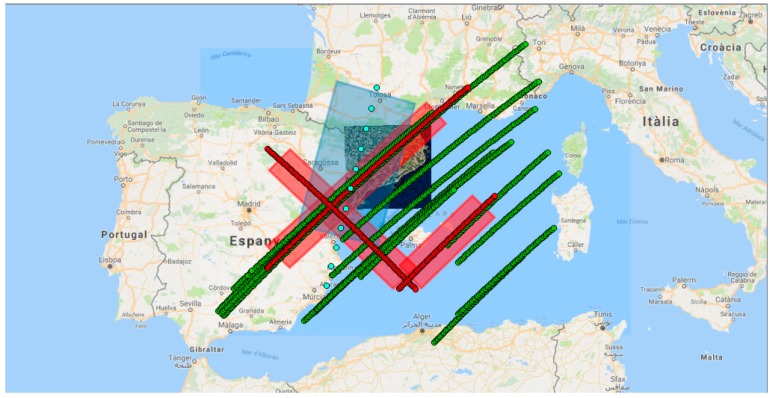
Simulation of the ^3^Cat-3track (red), the swath of the optic instrument (red blurred), possible specular reflection points (green), the Sentinel 2A/B pass closer to the Catalan territory (cyan), and the swath of the optic instrument of the Sentinel 2A/B (cyan blurred).

The scheduler handles the activity of the different subsystems and decides if the subsystems can be powered on, for how long, and over which regions. Scheduler activity has been defined as a combination of Target Areas (TAs) to be observed and Ground Stations (GSs) to contact the satellite. When the satellite flows over them, different subsystems are turned on: either the payloads to acquire data or the communication system to download data. The definition of these areas has a strong influence over the state of the satellite and the duty cycle of the different subsystems. In order to show representative simulation results, three TAs and three GSs have been considered in this mission analysis (Figure 3). The TA and GS over Europe overlap purposely because the UPC has a ground station located in the Observatori del Montsec [24], close to Barcelona. The scheduler decides which subsystem is active over which area, for how long, and under which budget conditions to achieve payload data fusion. In Figure 4, the data acquired by both payloads is depicted. The possibility to perform the data fusion with the optical payload on-board does not only depend on the simultaneous acquisition of data from both payloads. There are external factors, such as the meteorological conditions (i.e., clouds, fog) and the local time, that can disable the acquisition of optical data. Also, the specular reflection points can be located outside the optical swath of the camera, and therefore make data fusion impossible. To resolve this scenario, the track of the Sentinel 2A (equipped with a Multi-Spectral Imager) is also depicted to give an example of the possible use of another satellite’s data to achieve the final product of the ^3^Cat-3.

**Figure 5:**
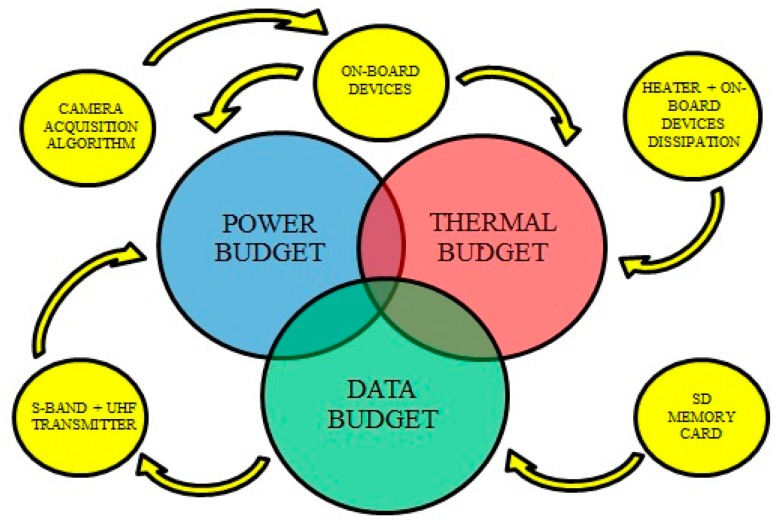
Conceptual diagram on the dependency of the activity of the different subsystems with the budgets that reflect the results of the simulation. UHF: ultra-high frequency; SD: secure digital.

Different scenarios can be dictated by the satellite’s internal state, as well as by external factors that should be approached by a different set of rules. The scheduler’s planning can never jeopardize the survival of the mission. The scheduler controls the activity and duty cycle of the different subsystems as sketched in Figure 5. The simulations presented in this report consider the following guidelines presented by priority-check order:

1. The thermal tolerance of all devices and materials on-board the satellite. The scheduler takes into consideration the energy dissipated in the form of heat by all devices on-board, as well as the Sun-eclipse periods experienced by the satellite. The heater will turn on if the temperature drops under a threshold and will not power off until the temperature reaches a certain level (5 °C and 8 °C, respectively). The batteries have the most restrictive temperature working range (between 0 °C and 45 °C); therefore, the hysteresis cycle that controls the heater prevents it from turning on/off constantly.
2. The impossibility to recharge the batteries in case of a total discharge as well as the maximum number of cycles of charge and discharge under different Depth of Discharge (DoD) levels as specified by the manufacturer (e.g., [25]).
3. The amount of data stored in the on-board memory. The scheduler is programed to give priority to the discharge of the data over the acquisition of new data when the amount of data stored on-board exceeds a certain limit. On the other hand, when the memory is below certain level (20% of the total storage maximum capacity) data acquisition has priority over the download of data.

## 3. Results

### 3.1. Mission Analysis

The mission analysis is carried out by monitoring the satellite’s capability to power up all subsystems (Power Budget), the heat balance (Thermal Budget), and the capability to store and download the acquired data (Data Budget). In order to have an overall vision of the satellite’s status the MOTS End-to-end Performance Simulator (MEPS) simulator has been developed [26]. In order to obtain consistent results, it is necessary to calculate all budgets simultaneously due to the interdependency of all variables that govern the system.

#### 3.1.1. Payload Analysis

It was decided that the ^3^Cat-3/MOTS mission would carry on-board two payloads: a multispectral optical sensor [27] in the VNIR (Visible and Near-InfraRed) [28,29] and a GNSS-Reflectometer. The final product will consist of data fusion from both payloads merging the multispectral image obtained from the optical sensor and the data collected by the geolocated reflectometer. This section details the performance of the optical sensor and the GNSS-Reflectometer from the point of view of the physical constraints and technical requirements to fulfill the mission statement.

• Optical Sensor

The selected COTS (Commercial off the Shelf) optical sensor and telephoto lens (Table 3) fulfill the mission statement described in Section 2 in terms of the Ground Sampling Distance (GSD) and swath. The study has thoroughly considered several COTS candidates for the optical sensor and lenses, but the decisive criterion was the solution adopted to provide multispectrality to the optical system with reliable technology. The standard solution of a filter wheel presents a problem for both the size of the wheel and the filter switch delay introduced into the acquisition of the multispectral image. The selected camera [29] has a set of up to two charge-coupled device (CCD) optical sensors with its own filters, solving the multispectrality issue. The size of the camera allows for a 75 mm optical focal length lens. The resulting GSD at nadir is calculated from the sensor’s pixel size (p), the focal length of the assembly (f), and the platform height (h) given the specifications of the manufacturer:

(1) $$\mathrm{GSD}=\frac{p\cdot h}{f}.$$

The predicted GSD values are only correct if the aperture of the optical system is large enough so as to satisfy the Rayleigh’s diffraction criterion. As the wavelength increases, the condition of the minimum aperture diameter (AP) of the optical system becomes more stringent:

(2) $${\mathrm{AP}}_{\min}=\frac{\lambda_{\max}\cdot h}{\mathrm{GSD}},$$

where λ_max_ is the longest wavelength among all bands. Assuming a narrow swath, it is directly calculated from the GSD obtained as:

(3) Swath≅#pixels·GSD,

where #pixels is the number of pixels in the cross-track direction. Another key parameter calculated after the swath is the Field of View (*FOV*). The lens manufacturer provides the *FOV* defined in the horizontal, vertical, and diagonal directions, which has to be larger than:

(4) $$FOV=2\cdot \arctan\left(\frac{\mathrm{swath}}{2\cdot h}\right),$$

so that the whole image is projected on the sensor. In order to measure the feasibility of the optical system in terms of image quality from the orbital configuration described, the signal-to-noise ratio (SNR) must also be calculated. The solar radiation spectrum at the top of the atmosphere (TOA) is not constant in all bands of interest; thus, the reflected electromagnetic (EM) wave received by the optical sensor has different signal power values for each band studied [5], (pp. 17–20).

(5) $$Signal\;Power=\frac{E_{\lambda_{0}}\cdot \tau_{\mathrm{atm}}^{2}\cdot \rho_{\lambda }\cdot {\mathrm{Area}}_{\det}\cdot {∆}_{\lambda }}{\pi \cdot G_{\#}},$$

where $E_{\lambda_{0}}$ is the Exo-Atmospheric Irradiance (EAI) in (W^2^/m^2^·nm), $\tau_{\mathrm{atm}}$ is the atmospheric transmission coefficient, $\rho_{\lambda }$ is the reflectance over the Earth’s surface, which depends on the albedo value, ${\mathrm{Area}}_{\det}$ is the area of the pixel in the detector provided by the manufacturer, ${∆}_{\lambda }$ is the receiver optical spectral width, and the G-number ($G_{\#}$), which includes the f-number ($f_{\#}$), characterizes the optical system:

(6) $$G_{\#}=\frac{1+4\times f_{\#}^{2}}{\tau_{\mathrm{opt}}\times \pi },$$

where $f_{\#}$ is defined as the ratio between the focal length and the aperture: $f_{\#}=f/\mathrm{AP}$, and $\tau_{\mathrm{opt}}$ is the optical transmissivity for all of the optical train (optical sensor plus lens). The other factor needed to compute the SNR is the Noise Equivalent Power (*NEP*), which is a measure of the goodness of the photodetector in terms of noise:

(7) $$NEP=\frac{\sqrt{2\cdot q\left(I_{ds}+F\cdot M^{2}\cdot I_{db}\right)\cdot B}}{ŋ\cdot \left(\frac{q\cdot \lambda }{c\cdot h}\right)\cdot M}.$$

In Equation (7), *q* is the electric charge of the electron (C), h = 6.63·10^−34^ J·S is the Planck constant, ŋ is the quantum efficiency, which changes for each band and it is particular for the optical sensor, $I_{ds}$ and $I_{db}$ are the surface and bulk dark currents, respectively (A), *F* is the excess noise factor (−), *M* is the multiplication factor of the avalanche diode, which in this case is *F* = *M* = 1, because there is no photo multiplication involved, and *B* (Hz) is the inverse of the integration time,

(8) $$B\geq \frac{1}{T_{integration}}=\frac{1}{\frac{GSD}{2\cdot V_{grd}}}=\frac{2\cdot V_{grd}}{GSD},$$

which is related to the $V_{grd}$ satellite’s ground speed and the *GSD* (Equation (1)).

Results are presented in Table 4 and fulfill the mission statement requirements. The following sections specify the design and simulated performance of all of the required subsystems needed to enable the payloads and the transmission of the data acquired to the ground stations.

• GNSS-Reflectometer

The GNSS payload of the ^3^CAT-3 is being developed in parallel with the ^3^Cat-4 ESA Fly Your Satellite project, in which the UPC NanoSat Lab [12] is also participating. This 1U CubeSat will carry a flexible microwave payload: an Automatic Identification System (AIS), a GNSS Reflectometer, and a microwave Radiometer [30]. The use of a software-defined radio (SDR) as a data logger is essential to reduce power consumption, the size of the payload, and cost. The scope of the ^3^Cat-3 is to perform data fusion between the optical payload and the GNSS-R data. The radiometer capabilities of the flexible microwave payload designed for the ^3^Cat-4 are being evaluated as a possible inclusion in the ^3^Cat-3 only if they do not jeopardize the development of the main mission in terms of power availability, data budget, and platform design. If the payload were to include a radiometer, the duty cycle of the down-looking antenna (GNSS receivers use GPS, Galileo, GLONASS, and Beidou systems) would increase and thus the power consumed would also increase. There are two possible configurations for the down-looking antenna with a minimum directivity of 12 dB: a 2 × 2 patch array, tested and integrated in the ^3^Cat-2 (in the ^3^Cat-2 it was a 2 × 3 patch array because all of the nadir-looking side was dedicated to the antenna), and a helix antenna, which is being developed for the ^3^Cat-4.

#### 3.1.2. Power Subsystem and Budget

The power subsystem consists of three separated main elements: the Electric Power System (EPS), the batteries, and the solar panels. An overall 80% efficiency has been considered for the Maximum Power Point Tracking (MPPT) and charger. The scheduler defines the set of rules under which the EPS will provide power to every subsystem on demand. On their behalf, each subsystem will try to be powered up when the satellite passes over certain defined areas (TA, GS, or during all orbit). Table 5 lists each subsystem, the regions over which they need to be powered on, and the typical and maximum power consumption. The battery heater’s schedule is controlled by the thermal budget, which monitors its temperature. The state of the battery heater responds to survival reasons, so it might need to be powered on over any area. Similarly, the ADCS needs to be powered on over TAs (for payload pointing accuracy) and GSs (for antenna pointing reasons), but the power peak when switching it on may indicate that it is preferable to keep the ADCS on during all orbit.

• Solar Panels

**Figure 6:**
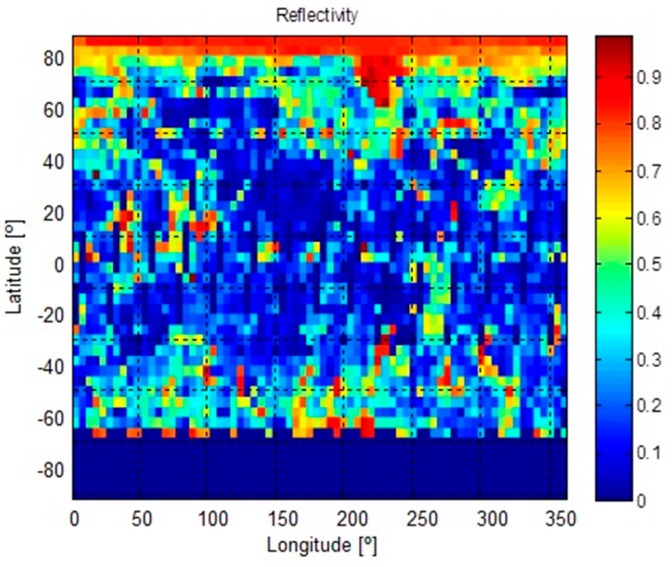
Reflectivity data from Earth’s surface (source TOMS, Total Ozone Mapping Spectrometer). The original matrix of 360 × 288 cells has been reduced in a factor of 4 to lighten the computing time. TOMS-EP measured total ozone by observing both incoming solar energy and backscattered ultraviolet (UV). By comparing the amount of backscattered radiation to observations of incoming solar energy at identical wavelengths, it is possible to infer Earth’s albedo.

**Figure 7:**
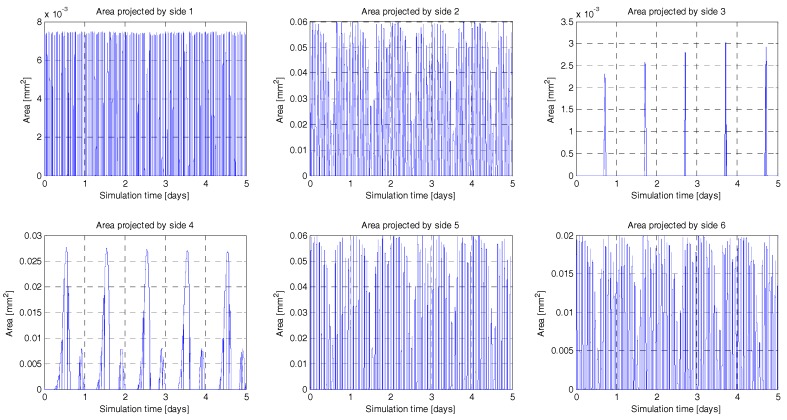
Instantaneous effective area exposed to the Sun of each side of the 6U CubeSat along 5 days of simulation. Each side of the CubeSat presents a different angle towards the Sun, which affects the collected energy.

The Indium Gallium phosphide/Gallium arsenide/Germanium (GaInP/GaAs/Ge) triple-junction cells solar panels considered have 30% efficiency [31]. In order to account for the total energy collected by the satellite, both the direct Sun radiation and the radiation scattered on the surface of the Earth (albedo radiation, Figure 6) have been considered changing along the track. The platform has been modelled as a ~10 × 20 × 30 cm^3^ parallelepiped so as to consider the incident angle between both radiation sources and the solar panels mounted on the sides (Figure 7). It is necessary to detail the orientation of the satellite to interpret the results of the simulation. The nadir direction is aligned with the −*z* axis and the linear velocity of the satellite is aligned with the +*y* axis.

• BATTERIES

Typically, the manufacturer provides the DoD [31] of the batteries as a maximum number of cycles of charge/discharge at 25% and 75% of the total charge (A·h) stored. The life cycle of the mission may depend, to a large extent, on the DoD policy. As specified by the scheduler, there are a few survival subsystems that must be powered on all the time, but others may not be activated due to low battery charge values.

• Power Budget

**Figure 8:**
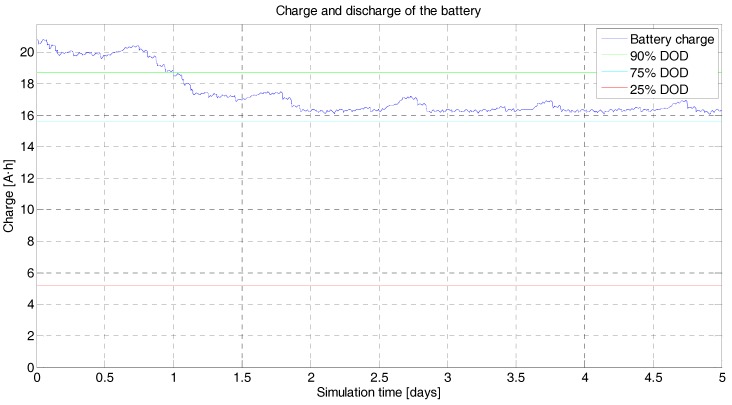
^3^Cat-3/MOTS power budget. In blue is the instantaneous charge of the batteries (A·h) during 5 days of simulation. After an initial transition period, the scheduler takes command and the budget is more predictable because the activity and duty cycles of the devices are firmly controlled. The green, blue, and red levels are at 90%, 75%, and 25% Depth of Discharge (DoD), respectively. The total battery capacity is 20.8 A·h, in a configuration of two modules of 10.4 A·h.

The power budget provides instantaneous battery charge throughout the simulation period (Figure 8). It shows the state of the batteries’ charge (A·h), already balancing the incoming/outgoing charge from the photovoltaic converter and to the subsystems. The eclipse periods, which account for approximately 30% of the orbit, are automatically treated by the scheduler, which denies all power demands from the subsystems that are not essential for survival. In the simulation, a maximum 20% usage of the total battery charge has been established in order to increase the lifespan of the satellite as much as possible.

#### 3.1.3. Thermal Budget

Temperatures in the thermosphere vary radically depending on the Sun’s illumination. The dependence on solar activity and the cycles of shadowing illumination every day form a highly variable thermal scenario. On the other hand, electronic devices have a well-defined temperature operating range (Table 6). The platform is exposed to high temperatures when in direct line of sight of the Sun and, if not properly defined, to very low temperatures when shadowed by the Earth. The simulation takes into account as heat sources the direct Sun radiation, the reflected radiation on the Earth’s surface (albedo radiation), the Earth’s radiation or Earthshine, and the internal heat dissipation of the electronic devices when they are turned on. On the other hand, the satellite itself radiates heat to space depending on the surface and emissivity of the solar cells and other materials.

**Figure 9:**
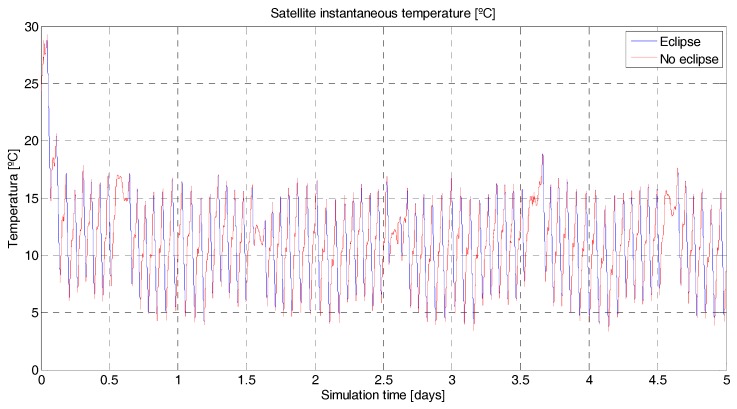
^3^Cat-3/MOTS thermal budget. In blue/red, instantaneous satellite temperature (°C) for a 5-day simulation. The red color shows the temperature when there is no solar eclipse. On the contrary, the blue color shows the temperature while in Sun eclipse periods. The satellite’s initial temperature has been set to 25 °C. There is a noticeable permanent regime temperature around 10 °C. The maximum temperature reached is approximately 27 °C and the minimum temperature 4 °C. The absorptance and emittance coefficients of the coating (polished beryllium) are 0.44 and 0.01, respectively.

**Figure 10:**
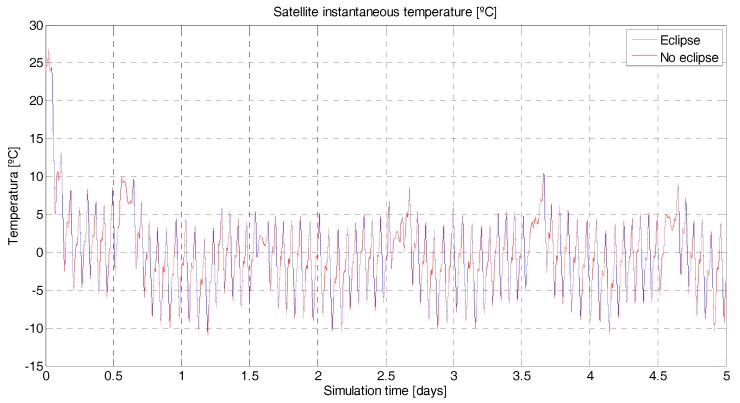
^3^Cat-3/MOTS thermal budget. In blue/red is the instantaneous satellite temperature (°C) for a 5-day simulation. The red color shows the temperature when there is no solar eclipse. On the contrary, the blue color shows the temperature while in Sun eclipse periods. The satellite’s initial temperature has been set to 25 °C. There is a noticeable permanent regime temperature around −2 °C. The maximum temperature reached is the initial temperature at 25 °C and the minimum is −11 °C. The absorptance and emittance coefficients of the coating (black paint) are 0.95 and 0.85m respectively.

To better control the satellite temperature, a coating should be applied to the surface of the satellite to vary the coefficient of absorptance and emittance. The satellite’s structure is covered with polished beryllium, which has an absorptance coefficient of α = 0.44 and an emittance coefficient of ε = 0.01 [23], (p. 363). This coating is will help to increase the satellite’s temperature that drops during eclipse periods (simulated instantaneous average temperature of the satellite, Figure 9). The polished beryllium has a medium absorptance coefficient, but an extremely low emittance coefficient. Another alternative explored was to apply a coating with higher absorptance in order to capture more heat, for example black paint (epoxy), α = 0.95 and ε = 0.85 [23], (p. 363). The simulations, however, show a much lower thermal budget due also to the higher emittance of the black paint (Figure 10).

As mentioned in Section 3. *C. Scheduler*, the thermal budget indirectly controls the battery heater which will power on independently of the scheduler if the temperature drops under certain levels to preserve the thermal range tolerance of the devices on-board.

#### 3.1.4. Data Handling and Budget

**Figure 11:**
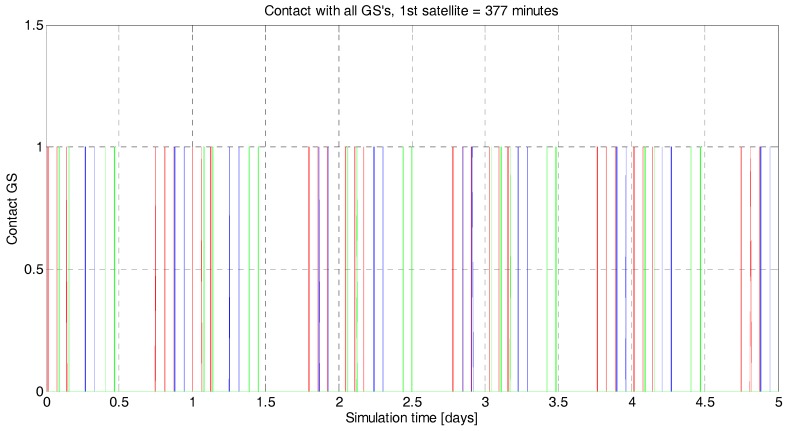
^3^Cat-3/MOTS contact time. In red, blue, and green is represented the contact with the ground stations described in Figure 2 (from left to right, respectively). In this particular case, the simulation lasts for 5 days and the total contact time is 377 min. This is approximately 5.3% of the orbital period. The minimum elevation over the horizon to stablish contact with the GS has been set to 10°.

The data download is performed with an S-band link when there is contact with a ground station and the scheduler powers on the communication subsystem. The amount of data acquired by the optical system can easily get out of hand if the duty cycle is not carefully controlled. The camera acquires images of 1.25 MP. With a digitalization of 12 bits/pixel, each image is in the order of 1.9 MB. Considering the size of the image (1296 × 966 pixels at approximately 26.6 m GSD), the necessary overlapping (5% to 15%) to ease the formation of the mosaic picture, and the ground speed of the satellite (7.06 km/s at an orbital altitude of 500 km), the optical system will generate roughly 4 Mbps of data (neither optical data compression, nor GNSS data acquisition have been considered). This amount of 4 Mbps of data generated at 100% of duty cycle is overwhelming. Typical commercial values of S-band link modules are less than 4 Mbps [31,32], and the average contact time with the GSs, located as described in Figure 2, is around 5.3% of the orbit (Figure 11). For the simulations, two scenarios have been considered for the S-band link speed: 0.1 Mbps and 0.5 Mbps. The contact time with the GSs is the same for all simulations: 5.3% of the orbital period.

**Figure 12:**
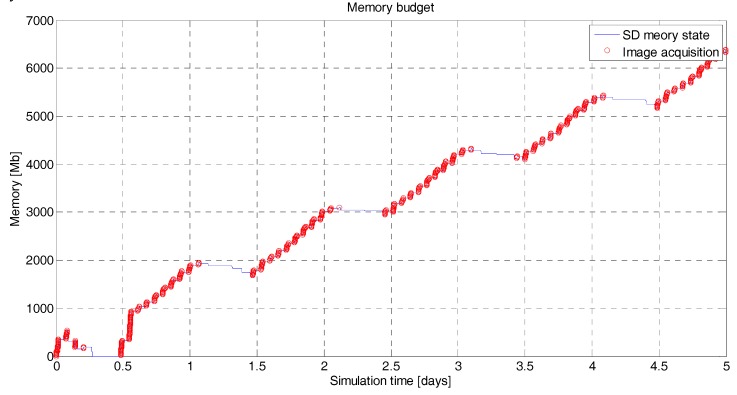
^3^Cat-3/MOTS data budget. In blue is the instantaneous state of the on-board memory storage in Mb. In red is the acquisition time of the payloads for a 0.1 Mbps downlink rate and five days’ simulation. The memory is rapidly getting full due to the inconsistent rate between acquisition and download capability. This effect can be corrected by reducing the acquisition area and/or increasing the transmission rate. In this simulation, the TAs and GSs are described in Figure 3.

**Figure 13:**
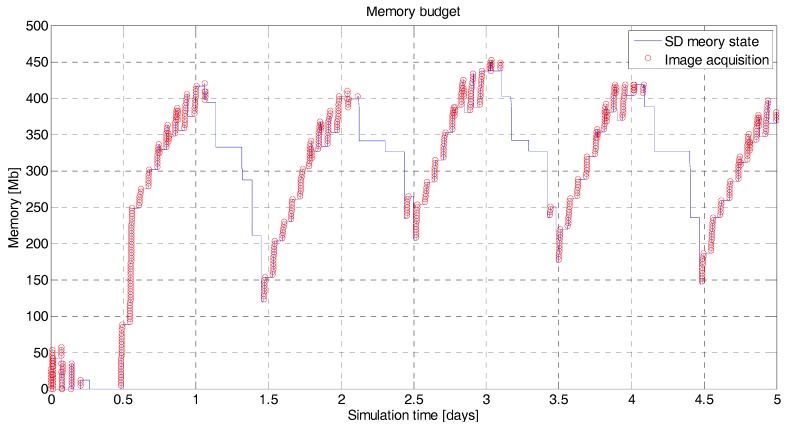
^3^Cat-3/MOTS data budget. In blue is the instantaneous state of the on-board memory storage in Mb. In red is the acquisition time of the payloads for a 0.5 Mbps downlink rate and five days’ simulation. The state of the memory is under control. The effect of Figure 12 has been corrected by increasing the transmission rate at 0.5 Mbps.

The data download is the final step of the main objective of the mission, so the scheduler gives priority to the data download over the acquisition of new data if the power available is disputed. It is assumed that the OBC has a storage limit of 2 GB, and two cases (a 0.1 Mbps data rate and a 0.5 Mbps data rate) have been assumed in order to perform the data budget. For the 0.1 Mbps downlink (Figure 12), the amount of data stored in the on-board memory is steadily growing. The transmission rate does not compensate for the data input flux, so given the acquisition configuration over the TAs, the mission will lose data acquired. This situation is corrected by increasing the download data rate to 0.5 Mbps (Figure 13). The output flux is now higher than the input flux so, all data acquired is rapidly downloaded to the GS’s.

## 4. Conclusions

A feasibility study for an optical/GNSS-R mission based on a 6U CubeSat has been performed and its summary is presented here. It includes the power, thermal, and data budget for a selected orbit.

For the optical payload, the longer the wavelength the stricter the Rayleigh’s condition is for the minimum aperture size (Equation (2)). On the other hand, a small GSD requires large focal lengths and small size detectors, which also increase the required aperture dimensions. The physical dimensions of the platform are the ultimate limiting factor [33]. This also applies to the physical dimensions of the GNSS-R antenna, which needs to have a directivity of at least 12 dB (either as a 2 × 2 patch antenna or as a retractable helix configuration as in ^3^Cat-4).

The power budget is, as usual, one of the mission’s bottlenecks, and the thorough configuration of the payload’s duty cycle will secure the success of the mission. With 31% of the orbital time in eclipse, the power available for all the subsystems and the two payloads will be very limited. The duty cycle will also have an impact on the temperature of the satellite; thus, the scheduler should consider that the priority in terms of the mission’s survival may come from the activity of the heaters and the contribution to raising the temperature of the active subsystems from dissipation. Last, but not least, the download of the data is of critical importance because the final product—soil moisture—will be obtained from two different payloads with differentiated duty cycles and with different amounts of data acquired (optical data is much larger than GNSS-R data). A stable and at least Mbps downlink is desirable to download the data acquired and not jeopardize the duty cycle of the payloads. In conclusion, the mission requires a delicate equilibrium between power, data, and thermal budgets, but is feasible and challenging at the same time, integrated within a 6U CubeSat and optical plus GNSS-R payloads.

## Figures and Tables

**Table 1:** Design requirements provided by the Institut Cartogràfic i Geològic de Catalunya (ICGC).

**Attitude Requirements**		
Pointing knowledge at nadir	120	arcsec
Pointing stability	20	arcsec/s
**Main Exploitation Requirements**		
Digitalization	12	bits
Data storage on board	>1	Gb
GSD at nadir pointing	<30	m
Swath at nadir pointing	>30	km
**Main Radiometric Budget**	**SNR (dB)**	**MTF (lp/mm)**
Blue band (440–510 nm)	35	25
Green band (520–590 nm)	35	25
Red band (620–680 nm)	35	25
Red edge band (690–730 nm)	35	20
NIR band (850–890 nm)	30	20
Extra band (if available)	30	20

GB: gigabytes; GSD: Ground Sampling Distance; SNR: signal-to-noise ratio; NIR: near infrared; MTF: Modulation Transfer Function.

**Table 2:** Reentry survivability analysis has been completed with DAS 2.02 [18]. The main subsystems and materials with their specific shape, thermal mass, size, quantity, and material type have been considered. All parts disintegrate between 71 km and 78 km height, therefore the casualty area and the kinetic energy are equal to 0.

Object Name	Sub Component Object	DemiseAltitude (km)
^3^Cat-3/MOTS	Chasis	73.7
	Solar panels	77.5
	ADCS subsystem	71.4
	Power Subsystem	75.9
	OBC	77.3
	Camera	77.1
	Lens	77.3

OBC: on-board computer; ADCS: Attitude Control and Determination System.

**Table 3:** Manufacturer’s specifications of both the optical sensor and the telephoto lens.

Optical Sensor	
Sensor tech	CCD
# pixels	1296 × 966
Pixel size (m)	3.75
Digitalization (bits)	8/12
Power consumption	12 VDC/8W
Shutter exposure (ms)	min. 6.5
LENS	
Focal length (mm)	75
Aperture (f/#)	f/2.8
Angle of view	D 12
Weight (g)	765
Diameter × length (mm)	36 × 64.3

CCD: charge-coupled device.

**Table 4:** Results of the mission analysis for the optical payload performance considering the Commercial off the Shelf (COTS) camera and lens specified in Table 3 and the orbital configuration described in Section 2.1.

Optical parameters	Bands performance				
Swath in (km):	34.7				
Max. aperture of the lens in (mm):	33.6				
Studied Bands:	475 (nm)	555 (nm)	650 (nm)	710 (nm)	870 (nm)
Aperture required for each band to satisfy Rayleigh criterion in (mm):	8.9	10.4	12.2	13.3	16.3
GSD for each band after Rayleigh criterion in (m):	26.8	26.78	26.78	26.78	26.78
SNR for each band in (dB):	37.3	40.4	41.2	41.6	36.9

**Table 5:** The different subsystems will try to power on over different regions. The scheduler is in charge of checking the state of the satellite to allow or deny the activation of all non-survival subsystems. Green—attempt to power up; Yellow—powered up under survival conditions and power save mode; Red—no attempt to power up.

	Consued Power		TA	GS	All Orbit
	Typical (W)	Max. (W)			
Optical payload	-	6			
GNNS-R payload	1.5	2			
Battery heater	0.5	2.5			
ADCS	0.5	2.5			
S-band TX	8	12			
VHF TX	2.9	3.1			
Primary OBC	2.3	2.3			
Secondary OBC	0.2	0.9			

GNNS-R: Global Navigation Satellite Systems Reflectometry.

**Table 6:** Operational temperature (°C) of all devices on-board the ^3^Cat-3/MOTS sensible of malfunctioning due to temperature changes. Higlighted in red, the batteries, are the most restrictive temperature conditions’ device.

	Operational Temperature (°C)	
Device	min.	max.
6U Chasis	−40	85
Solar panels	−40	85
EPS	−40	125
Batteries	0	45
ADCS	−40	80
OBC	−40	60
S-band TX	−40	85
Optical payload	−40	60

EPS: electric power system.
